# Polygenic risk scoring of human embryos: a qualitative study of media coverage

**DOI:** 10.1186/s12910-021-00694-4

**Published:** 2021-09-18

**Authors:** Tiny Pagnaer, Maria Siermann, Pascal Borry, Olga Tšuiko

**Affiliations:** 1 Department of Public Health and Primary Care, Centre for Biomedical Ethics and Law, KU Leuven, Leuven, Belgium; 2 Laboratory for Cytogenetics and Genome Research, Department of Human Genetics, Centre for Human Genetics, KU Leuven, Leuven, Belgium

**Keywords:** Ethics, Qualitative analysis, Media coverage, Preimplantation genetic testing, Polygenic risk scores, Embryo polygenic profiling, PGT-P

## Abstract

**Background:**

Current preimplantation genetic testing (PGT) technologies enable embryo genotyping across the whole genome. This has led to the development of polygenic risk scoring of human embryos (PGT-P). Recent implementation of PGT-P, including screening for intelligence, has been extensively covered by media reports, raising major controversy. Considering the increasing demand for assisted reproduction, we evaluated how information about PGT-P is communicated in press media and explored the diversity of ethical themes present in the public debate.

**Methods:**

LexisNexis Academic database and Google News were searched to identify articles about polygenic embryo screening. This led to 535 news articles. 59 original articles met the inclusion criteria. Inductive content analysis was used to analyse these articles.

**Results:**

8.8% of articles gave embryo polygenic scoring a positive portrayal, while 36.8% expressed a negative attitude. 54.4% were neutral, mostly highlighting limited practical value of the technology in in vitro fertilization settings. We identified five main ethical themes that are also present in academic literature and the broader debate on reproductive technologies: a slippery slope towards designer babies, well-being of the child and parents, impact on society, deliberate choice and societal readiness.

**Conclusions:**

Implementation of embryo polygenic profiling engenders a need for specific recommendations. Current media analysis discloses important ethical themes to consider when creating future guidelines for PGT-P.

**Supplementary Information:**

The online version contains supplementary material available at 10.1186/s12910-021-00694-4.

## Background

Over the past thirty years, there have been many technological breakthroughs in the field of preimplantation genetic testing (PGT). Currently, application methods are classified into (1) preimplantation genetic testing for monogenic disorders, or PGT-M, (2) preimplantation genetic testing for aneuploidy, or PGT-A, and (3) preimplantation genetic testing for structural rearrangements, or PGT-SR, designed to help carriers of balanced translocations to achieve a successful pregnancy. Today PGT-M is performed to select against any Mendelian hereditary conditions with a known cause, including autosomal-recessive, autosomal-dominant and X-linked disorders. Recent implementation of genome-wide genotyping and haplotyping methods also made PGT-M workflows more generic and standardized, precluding the need to design family or locus-specific protocols [1–5]. In contrast to PGT-M and PGT-SR, where mostly young fertile couples are undergoing treatment to avoid embryos with pathogenic variants or unbalanced karyotypes, PGT-A is offered to couples with fertility issues. Since preimplantation embryos are burdened with aneuploidy, it was suggested that PGT-A could improve in vitro fertilization (IVF) success rate per embryo transfer in sub-fertile couples. Despite major controversy [6–8], PGT-A has been implemented in many IVF centres worldwide to select against embryos with abnormal karyotype.

Currently performed PGT procedures are generally accepted as an embryo selection tool, with most countries having their own legal and regulatory frameworks [9, 10]. In some countries in Europe the use of PGT is rather restricted, while the policies for PGT in the United States are more permissive, stimulating its use for controversial indications (e.g. social sex selection or HLA typing) and reproductive tourism [11, 12]. Furthermore, existing loopholes in established restrictions allow surpassing of current norms of conventional embryo screening for monogenic diseases or chromosomal abnormalities. While the first clinical application of CRISPR technology on human embryos was heavily criticized by the scientific community, calling for a moratorium on embryo gene editing for clinical purposes [13], the use of polygenic risk scoring for embryo selection recently became a reality [14, 15]. Derived from large-scale genome-wide association studies (GWAS), polygenic risk scores (PRS) estimate the cumulative effect of numerous genetic variants to assess an individual’s susceptibility to complex diseases, such as cardiovascular diseases or type 2 diabetes [16]. The main idea of PRS is to leverage precision medicine, by stratifying individuals into low- or high-risk groups based on their genetic susceptibility to certain diseases. The ability to genotype embryos allowed translating this concept to PGT for polygenic disorders, or PGT-P, to perform embryo profiling for a variety of complex diseases and traits, including intelligence [14, 15]. The novel application was subsequently commercialized by Genomic Prediction Inc., a US-based company, which offers couples embryo polygenic risk estimation for complex traits, including intellectual disability [17].

The use of polygenic scoring for embryo selection might become a tempting add-on for couples entering assisted reproduction programs. Upon its announcement, PGT-P immediately gained extensive media coverage, raising medical, scientific and ethical issues. Given that media sources play an important role in shaping public opinion of scientific and healthcare advances, it is important to evaluate how information about PGT-P is communicated to the general public. Therefore, our study aimed to explore the ethical and social themes present in the public debate as reported in press media. We performed a qualitative study using inductive content analysis to garner insights about the portrayal of PGT-P in news articles.

## Methods

### Data collection

We used the LexisNexis Academic database and Google News to find relevant news articles that covered the topic of polygenic risk scoring in embryos (PGT-P). The search term “Genomic Prediction” with exact word combination and capital letters was used to recover news articles published between May 1st, 2017 and June 1st, 2019. This period coincides with the foundation date of Genomic Prediction Inc., the U.S. based company that was the first to offer PGT-P, and the publication of the first clinical case of polygenic risk scoring in human preimplantation embryos [14]. Articles written in Dutch, English, French and German were included to get a broad view on ethical aspects of PGT-P in diverse countries. The initial search yielded 535 news articles (202 in LexisNexis and 333 in Google News, respectively). Articles with content irrelevant to the ethics of PGT-P (e.g. economic reports of the company) were excluded from the analysis. This led to inclusion of 88 articles.

### Data analysis

We used inductive content analysis to analyse the included articles. This form of analysis, also referred to as conventional content analysis, is appropriate for analysing documents when limited theory and research exists about the topic. An inductive approach is used: instead of using preconceived categories, categories are directly based on the data [18]. The original news articles that fit the selection criteria were therefore openly coded. Initial code labels were taken directly from the text and a preliminary coding scheme was developed upon repeated reading of the material. We performed multiple rounds of coding. With each round, we organized the number of codes into a smaller subset of codes. These codes were then redistributed to achieve a final branched coding scheme with sub-codes for each category. No coding software was used for the data analysis. Additionally, the content of each article was assessed for news portrayal tone and classified as (1) positive, if only benefits were discussed, (2) negative, if only concerns were raised and (3) neutral, if multiple views were given and/or there was no specific emphasis on positive or negative sides. Coding was done by two researchers (TP and OT) independently in order to minimize bias. In case of disagreements, researchers discussed until consensus was reached.

## Results

**Fig. 1:**
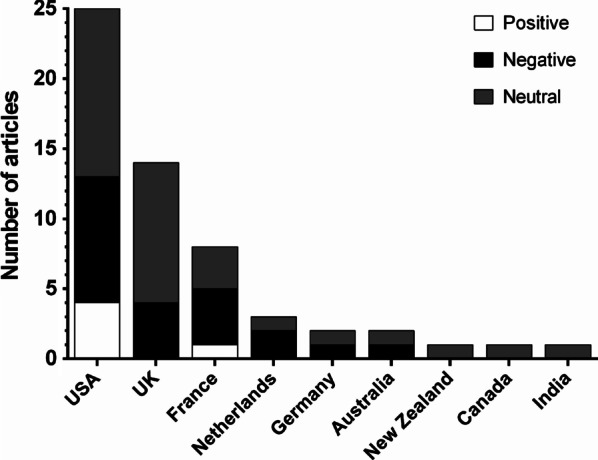
Tone of articles per country

We retrieved a total of 88 articles, out of which 59 were original and the remaining 29 were republications by other news sources (Additional file 1: Table S1). Most original articles were published in United States-based media (43%, Fig. 1). Half of the articles (54.4%) had a neutral tone: while acknowledging the developmental initiative, they also highlighted practical and technical limitations of polygenic scoring in IVF settings. One third of articles (36.8%) expressed a negative attitude towards PGT-P and only 8.8% saw it as a positive advancement in the field (Fig. 1). Articles were published in various media sources, such as serious broadsheet newspapers, tabloid newspapers, science magazines and religious magazines. Next, we extracted five main themes with respective subthemes that addressed ethical aspects of performing PGT-P (Table 1). Although PGT-P indications include various complex trait diseases, such as diabetes or cardiovascular diseases, the main issue discussed in the news articles concerned embryo screening for intelligence.

**Table 1:** List of themes and sub-themes

Theme	Subtheme
Theme 1. A slippery slope towards designer babies	Concern about a slippery slope Concern about eugenics Reassurance about the misuse of PGT-P Recurring argument Limited possibilities and usefulness
Theme 2. Well-being of the future child and parents	Positive impact on well-being of the future child Moral duty to improve quality of life of future child Concern about well-being of the child Value conflict for parents Positive impact on well-being of the parents Pressure to use artificial reproduction
Theme 3. Impact on society	Socio-economic impact Concern about economic incentives Issue of discrimination and stigmatization Attitude of society towards disabled people More equality in society Individual rights vs. needs of society Relative significance of individual characteristics
Theme 4. Deliberate choice	Positive impact on deliberate choice What lives are worth living? Concern about deliberate choice Dilemma of one remaining embryo Reassurance about deliberate choice
Theme 5. Societal readiness	Concern about unregulated commercialization Need for ethical debate Need for protection Lack of scientific validity Drawback of polygenic risk scoring Unwanted consequences

### Theme 1: a slippery slope towards designer babies

The most common concern present in the news coverage was the potential use of PGT-P for non-medical traits, such as hair or eye colour. This was often described as a slippery slope towards designer babies (Q1, Additional file 1: Table S2). This "slippery slope" was mentioned in 50 out of 59 included articles (84.7%). The negative reaction was associated with eugenic practices and many feared that the technique would be used for genetic enhancement (Q2–Q3, Additional file 1: Table S2). At the same time, many articles cited Genomic Prediction founders, who reassured that the company will always consider the ethical standards of the community and will not use the technique for controversial reasons (Q4–Q5, Additional file 1: Table S2). Moreover, recurring arguments about designer babies and eugenics highlighted that this type of ethical debate appeared with the introduction of every new reproductive technology (Q6–Q7, Additional file 1: Table S2). From a practical side, various articles mentioned that PGT-P provides very few benefits: considering that as IVF treatment yields a relatively small number of embryos and that children from  the same parents show little significant genetic variation, the number of options to choose from will be limited. As such, the use of PGT-P will only have limited consequences, making the fear of a slippery slope unjustified.

### Theme 2: well-being of the child and parents

Positive elements of PGT-P for well-being of children and parents appeared in 55.9% of articles. Several articles emphasized the positive features of PGT-P, such as reducing the risk of certain diseases and the possibility of improving future children’s health and well-being (Q1–Q2, Additional file 1: Table S3). Others noted that the new technique will lead to more successful pregnancies and better IVF care (Q3, Additional file 1: Table S3). Furthermore, by improving the child’s well-being, the parents’ emotional and financial burden of care is also moderated (Q4, Additional file 1: Table S3). Because of these positive outcomes, some argued that if we agree that parents are obliged to promote their children’s life quality, they should be able to use this technique to ensure best quality of life for their children (Q5, Additional file 1: Table S3).

Opposing views feared that children might feel pressured due to high expectations, which could consequently bring a great deal of emotional frustration and harm (Q6, Additional file 1: Table S3). The parents might be disappointed in their future child, if the child does not meet their standards, especially when they spent money for the “best” embryo. In addition, even if there is no medical reason *stricto sensu*, future parents may consider using IVF to have healthier children, despite it being time-consuming, invasive and expensive (Q7–Q8, Additional file 1: Table S3).

### Theme 3: impact on society

71.2% of articles referred to the impact of the technology on society. This referred to impact in terms of economics, equity, discrimination, individual rights and the health of society. According to some authors, embryo selection might have a positive socio-economic impact: social costs would lower, economic performance would augment, and medical resources could be allocated more efficiently in the future. At the same time, the importance of economic incentives was questioned. Although hypothetically populations could be altered in favour of higher economic performances by continuously selecting for higher IQ, high economic performance and improved healthcare might not be the only driving force on the market. Selection could for example also be requested for other traits such as skin colour (Q1, Additional file 1: Table S4). Hence, many articles highlighted the difficulty of solving the dilemma between what is ethically desirable and economically advantageous for our current and future society.

With regards to equity, there were concerns that access to PGT-P would be limited to those who could pay for it. Consequently, this could further increase the already existing social gap, with the wealthy class trying to improve the intelligence and health of their offspring (Q2, Additional file 1: Table S4). Another concern was the message we give as a society to people with disabilities. By performing trait selection at embryo level, people with “undesirable” traits could be regarded as less valuable than the ones without disabilities or diseases. One author argued that by eliminating disabilities and biological diversity, we risk losing differences between people, and thus also sources of creativity (Q3, Additional file 1: Table S4).

Additionally, potential unequal access to PGT-P raised questions about the balance between what is good for society and what is good for the individual. Promoting individual welfare might not be good for society, because it could create a societal division between those who have access to the test and those who do not (Q4, Supp. Table S4). At the same time, access to the test would depend on differences between countries’ healthcare systems, which is part of a broader debate. One author argued that using PGT-P for selecting embryos is not very different from what we already do in everyday life: after birth, we try to promote advantageous characteristics like intelligence by, for example, sending children to private education. The same author also pointed out that a strong society is determined by collective intelligence, which comes as a result of many people working together, rather than reliance on the individual “braininess”.

As described in Theme 1, the possibility of reviving eugenics was seen as a major concern. However, some argued that from a societal point of view, improving humanity is not necessarily a frightening prospect: we might take control of our evolution by curtailing some of the genetic diversity and thus lowering the incidences of disease.

### Theme 4: deliberate choice

Different authors suggested that PGT-P could benefit reproductive liberty, as it would enable individuals to make informed decisions about the pregnancy. This theme was present in almost half (45.8%) of articles. If one is already performing PGT-M for embryo selection, polygenic risk scores screening could be added without the need for an additional biopsy. Also, PGT-P could potentially help prospective parents going through IVF treatment to prioritize embryos for transfer, based on these genetic scores (Q1–Q2, Additional file 1: Table S5). At the same time, freedom of choice could also lead to existential questions, such as what lives are worth choosing and what would be the impact of such decisions (Q3, Additional file 1: Table S5).

According to others, the technique might contradictorily lead to less reproductive liberty, because it might become the parents’ moral duty to select the best possible embryo (Q4, Additional file 1: Table S5). Similarly, doctors might have an ethical responsibility to report possible negative outliers (Q5, Additional file 1: Table S5). Couples may also face a dilemma, if they have only one available embryo for transfer and that embryo has undesirable scores. In that case, reproductive liberty could decrease.

### Theme 5: societal readiness

Another significant issue raised by media sources concerns premature availability of the test on the market. About two-thirds (67.8%) of articles referred to societal readiness for the technology. Many emphasized the need for a broad ethical debate, stressing the urge for regulation to protect against the use of uncertain and debatable PGT applications (Q1–3, Additional file 1: Table S6). According to different articles, variable PGT regulation between countries could lead to medical tourism, if one country would allow something another does not.

Various authors also questioned scientific evidence underlying PGT-P. First, the test would not be reliable enough due to moderate accuracy of PRS calculation in embryos, considering the limited starting DNA material (Q4, Additional file 1: Table S6). Second, polygenic traits can be determined by many different genes and selecting for few individual genes, associated with a certain trait, would be too limited to use them in predictions (Q5, Additional file 1: Table S6). In addition, upbringing, lifestyle and environmental factors play a major role in determining a trait (Q6, Additional file 1: Table S6). Third, PRS calculations operate on a population level, but they cannot be extrapolated to an individual for direct risk assessment and current statistical predictors are not valid for people of non-European descent (Q7–Q8, Additional file 1: Table S6). This drawback could be especially relevant to the equity concerns addressed in Theme 3. Finally, the causal relationship between genes and a trait often remains obscure. For example, genes associated with higher intelligence can overlap with genes for autism, hence selecting for high IQ could lead to adverse consequences (Q9, Additional file 1: Table S6). This led to concerns about genetic diversity and unforeseen negative impacts if we would try to promote certain traits.

## Discussion

Novel genetic technologies often spark a public debate, regarding their utility and appropriateness of use. For example, while CRISPR technology for developing treatment for genetic or complex diseases is portrayed by media as beneficial, its use on human embryos is regarded as problematic [19]. We observed a similar trend in our analysis: in the context of precision medicine, the development of PRS for adult screening is typically considered a positive healthcare trend [20]; however, it raised several ethical issues when applied in embryos. These mainly concerned embryo selection for non-medical traits, the well-being of future child and parents, principles of procreative beneficence and procreative altruism, reproductive liberty and scientific validity of the test. The wary perception of PGT-P is not surprising, considering the historically polarized view on embryo screening for adult-onset diseases and limited support for embryo selection for non-medical or cognitive traits both from the public [21–23] and healthcare professionals [24].

One of the biggest critiques raised in the news articles is the lack of solid scientific evidence for PGT-P beneficence, claiming the test to be technically flawed. For this reason, the implementation of the technology in clinical practice was considered premature. Many of the discussed issues that can impact polygenic scoring - such as environmental risk factors, stratification effect and lack of predictive power in people of non-European ancestry - have been previously acknowledged by the scientific community [25–27]. A recent simulation study also demonstrated a limited gain in value when selecting embryos for height and IQ [28]. Furthermore, ethical issues arise around the possibility of using PGT-P for psychiatric traits and/or "desirable traits" such as height and "intelligence". Some of the recently published works also address the issue of phenotypic variation of screened conditions and pleiotropy, which means that a certain polygenic score for one trait has a correlation with another polygenic trait that the couple does not want to select for. As a consequence of these issues, the implications of PGT-P might be difficult to understand for couples who consider using the technology, and the increased options with PGT-P could lead to a "paradox of choice", which in turn could reduce reproductive autonomy.[29–31].

However, as we have shown, despite technical and practical pitfalls associated with PGT-P, some authors of media articles strongly promoted its use. They emphasized that the ability to select the “best” embryo to prevent diseases and avoid unwanted traits is an important incentive that will have positive impacts both on an individual level and for society as whole. Regarded as “scientific hype”, these possibly unreliable beneficial claims could mislead the general public, creating false expectations. In the same vein, the negative portrayal of PGT-P possibly exaggerated some of the issues associated with the test. However, given the current uncertain value of PGT-P in IVF settings, unreasonable sensationalism (both positive and negative) of scientific news together with generally poor understanding of technological possibilities could consequently lead to loss of public trust in science [32]. Importantly, half of the articles had a neutral portrayal of the topic, which often reduced both the exaggerated benefit and fear associated with “designer babies”. Balanced reporting of pros and cons can be instrumental in scientific and ethical debates in IVF, especially considering the growing commercialization of reproductive genetic technologies. In a highly competitive environment, fertility centres frequently offer patients different IVF add-ons without regulatory oversight, even in the absence of evidence-based clinical value [33, 34]. Hence, providing accurate and reliable information is crucial for decision-making with respect to patient’s reproductive liberty. It would also allow to protect vulnerable patients from excessive financial and psychological burdens associated with the treatment [35].

Several themes observed in our study were recently discussed by the scientific community in the context of embryo gene-editing [36]. In addition, they were present in the report “Genome editing and human reproduction: social and ethical issues” published by the Nuffield Council on Bioethics [37]. However, this report specifically targeted gene editing, hence it cannot be fully extrapolated to PGT-P due to the different nature of these technologies. Similarly, current PGT-M and PGT-A best practice guidelines and recommendations, released by major international societies, such as the PGD International Society (PGDIS) [38], the American Society for Reproductive Medicine (ASRM) [39] and the European Society of Human Reproduction and Embryology (ESHRE) PGT consortium [40, 41] do not consider the expanded possibilities that might be generated by the arrival of polygenic scoring. Rather than selecting the “best” embryo, PGT-P has the potential to prioritize embryos for transfer and/or reduce the risk of having a child with severe clinical phenotypes. Thus, questions arise whether it is appropriate to use PGT-P for embryo selection; if so, for which diseases or traits, and who should be able to make such decisions.

Interestingly, in some other articles that reported on media coverage of technologies related to genetics and reproduction, the perception found in media articles was more positive. In the studies on newspaper representations of non-invasive prenatal testing in the United States [42] and the United Kingdom [43], authors concluded that the benefits of the technology are more often described than the harms, which could lead to unrealistic expectations of the test. In studies on media coverage of social egg freezing [44] and Angelina Jolie’s preventive bilateral mastectomy and BRCA1/2 testing [45], a similar positive attitude to the technologies is found. Perhaps the focus on non-medical traits and possible lack of scientific evidence with PGT-P could be a reason for this difference in attitudes.

Our study has limitations. Inherent to any qualitative methodology, coding was subjective, although we tried to minimize the bias by involving multiple researchers to deduce codes and reach a consensus. In addition, we did not check the validity of the statements made in media coverage, as it was not our goal to evaluate the accuracy of reporting, but to show how media broadcasts PGT-P and thus can shape public opinion. Furthermore, the only search term we used was “Genomic Prediction”. This search term was chosen because at the time of the search this was the emerging company offering PGT-P and the company being covered in news articles, which is why it most likely covers most news articles at the time. However, it is possible that we missed out on some news articles by only using one search term. Our analysis also only focused on published news articles, whereas a lot of information is also shared using videos, podcasts or social media. Information presented on radio or TV was also not included. In addition, multiple regions remain uncovered, which might introduce a cultural bias in our selection of ethical themes. Hence, our results may not be fully generalizable, and more research is warranted to understand public views (including of stakeholders and prospective patients) on this matter.

## Conclusions

In conclusion, current media analysis on polygenic risk scoring in embryos reflected most of the themes and issues present in scientific and ethical literature. Scientists, doctors and ethicists need to engage with the general public to (1) further explore the present ethical themes, (2) improve science coverage in media publications and (3) ensure patients receive accurate information for informed decision-making. In addition, the disclosed themes should be considered when creating future recommendations about innovations in PGT, in particular PGT-P.

## Supplementary Information

**Additional file 1: Table S1**. List of primary news articles’ where the included media articles are listed. It also includes tables where the relevant quotes relating to each theme are listed, namely. **Table S2.** Quotes from Theme 1: A Slippery Slope Towards Designer Babies. **Table S3.** Quotes from Theme 2: Well-being of the Child and Parents. **Table S4.** Quotes from Theme 3: Impact on Society. **Table S5.** Quotes from Theme 4: Deliberate Choice. **Table S6.** Quotes from Theme 5: Societal Readiness.

## Data Availability

All data generated or analysed during this study are included in this published article [and its Additional file 1].

## Abbreviations

ASRM: American Society for Reproductive Medicine
ESHRE: European Society of Human Reproduction and Embryology
GWAS: Genome-wide association studies
IVF: In vitro fertilization
PGT: Preimplantation genetic testing
PRS: Polygenic risk scores
PGDIS: Preimplantation Genetic Diagnosis International Society
PGT-A: Preimplantation genetic testing for the presence of aneuploidy
PGT-M: Preimplantation genetic testing for monogenic conditions
PGT-P: Preimplantation genetic testing for polygenic conditions
PGT-SR: Preimplantation genetic testing for structural chromosomal rearrangements
