# Supplementary material for: Polygenic risk scoring of human embryos: a qualitative study of media coverage
Source: BMC Med Ethics. 2021 Sep 18;22:125. doi: 10.1186/s12910-021-00694-4 (PMC8449454; doi:10.1186/s12910-021-00694-4)
Supplement: Supplementary file 1 — Additional file 1: Table S1. List of primary news articles’ where the included media articles are listed. It also includes tables where the relevant quotes relating to each theme are listed, namely. Table S2. Quotes from Theme 1: A Slippery Slope Towards Designer Babies. Table S3. Quotes from Theme 2: Well-being of the Child and Parents. Table S4. Quotes from Theme 3: Impact on Society. Table S5. Quotes from Theme 4: Deliberate Choice. Table S6. Quotes from Theme 5: Societal Readiness. [file 12910_2021_694_MOESM1_ESM.docx]

**Supplementary tables**

Supplementary Table S1. List of primary news articles

| **Publication date** | **Source** | **Title** |
| --- | --- | --- |
| 27 July 2017 | Targeted News Service | Genomic Prediction Moves to CCIT, Cites Array of Resources, Ideal Location As Factors |
| 27 July 2017 | States News Service | ↻ Genomic Prediction Moves to CCIT, Cites Array of Resources, Ideal Location As Factors |
| 1 November 2017 | MIT Technology Review | Eugenics 2.0: We’re at the Dawn of Choosing Embryos by Health, Height, and More |
| 2 November 2017 | Next Big Future | More advanced genomic screening for embyro selection for IVF |
| 14 December 2017 | C Net France | Procréation du futur: bientôt des bébés à la carte? |
| 16 December 2017 | The Spectator | The ‘designer baby’ myth; Even if we could create super-bright offspring, there would be no demand for them |
| 11 January 2018 | Rockland Country Times | Ombudsman alert: will you be among the first to pick your kids’ genes? |
| 10 April 2018 | Reason | Should the Government Ban Parents Using IVF from Picking Their Kid’s Eye Color? |
| 23 May 2018 | Le Monde | Bientôt des bébés à la carte? |
| 3 October 2018 | The Wall Street Journal | Is It Ethical To Choose Your Baby’s Eye Color? |
| 9 October 2018 | USA Today Network | Want a designer baby? NJ lab closer to making science fiction a reality |
| 14 November 2018 | New Scientist | Choose your child’s intelligence |
| 15 November 2018 | New Scientist | ↻ Exclusive: A new test can predict IVF embryos’ risk of having a low IQ |
| 26 November 2018 | The Times of India | ↻ Now, choose your child’s intelligence |
| 15 November 2018 | Numerama | Fécondation in vitro: aux États-Unis, un test génétique estime­rait le futur QI d’un embryon |
| 15 November 2018 | Daily Mail | Designer baby fears grow as experts slam ‘repugnant’ new test that can predict an IVF embryos’ risk of having a low IQ |
| 15 November 2018 | Futurism | New test predicts how smart babies will be before they’re born |
| 16 November 2018 | Medical Xpress | Progress in genetic testing of embryos stokes fears of design­er babies |
| 17 November 2018 | Pharma & Healthcare Monitor Worldwide | ↻ Progress in genetic testing of embryos stokes fears of designer babies |
| 19 November 2018 | Yerepouni Daily News | ↻ Progress in genetic testing of embryos stokes fears of designer babies |
| 16 November 2018 | The Sun | WT(IV)F? New test means parents could soon pick kids via IVF ‘partly based on intelligence’ |
| 16 November 2018 | The Times | New test can predict intelligence in embryos |
| 17 November 2018 | The Press | ↻ Test can predict intelligence in embryos |
| 16 November 2018 | earth.com | Embryo screening for IQ is now possible, but is it ethical? |
| 17 November 2018 | iatranshumanisme.com | Choisissez l’intelligence de votre enfant |
| 16 November 2018 | National Post | Colby Cosh: Gene scoring and superbabies on four legs — and two |
| 16 November 2018 | Postmedia Breaking News | ↻ Colby Cosh: Gene scoring and superbabies on four legs — and two |
| 17 November 2018 | National Post | ↻ Superbabies are no cause for alarm |
| 17 November 2018 | The Times | Master race dystopia is closer than we think |
| 19 November 2018 | Gènéthique | Vers le tri des embryons selon leur qi? |
| 19 November 2018 | The Guardian | Super-smart designer babies could be on offer soon. But is that ethical? |
| 19 November 2018 | BioNews | Genetic test to screen embryos for low intelligence developed in US |
| 20 November 2018 | LifeNews | New Genetic Test Could Result in Killing Unborn Babies Deemed “Less Intelligent” |
| 22 November 2018 | RTL | Un test génétique pour détecter l'intelligence de son futur bébé ? |
| 22 November 2018 | Futura | Bientôt des bébés sur mesure nés d’une FIV? |
| 22 November 2018 | Catholic News Agency | Embryonic IQ tests could ‘screen’ for less intelligent children, firm says |
| 22 November 2018 | Financial Times | Profiling for IQ opens new uber-parenting possibilities |
| 23 November 2018 | Financial Times | ↻ Intelligence profiling opens up uber-parenting potential |
| 24 November 2018 | de Volkskrant | Omstreden IQ-test embryo’s ligt klaar |
| 28 November 2018 | geek.com | New Genetic Test Lets IVF Patients Screen Out Risky Embryos |
| 28 November 2018 | The Next Web | Controversial new test could be used to screen embryos for intelligence |
| 29 November 2018 | IT Next | ↻ Controversial new test could be used to screen embryos for intelligence |
| 1 December 2018 | Forbes | Parents Can Already Use Genetics To Select For Healthier, Maybe More Intelligent, Babies |
| 2 December 2018 | Psychology Today | The Future of In-Vitro Fertilization and Gene Editing |
| 2 December 2018 | Welt | Werden Embryonen bald nach Intelligenz aussortiert? |
| 5 December 2018 | Bilanz | ↻ Werden Embryonen bald nach Intelligenz aussortiert? |
| 3 December 2018 | Outer Places | Even Without CRISPR, Parents Can Genetically Tailor Smarter IVF Babies |
| 10 December 2018 | The Conversation | Those designer babies everyone is freaking out about — it’s not likely to happen |
| 10 December 2018 | Middle East and North Africa Financial Network | ↻ Those designer babies everyone is freaking out about — it’s not likely to happen |
| 12 December 2018 | The Wire | ↻ Creation of Designer Babies Is Limited by Biology, Not Technology |
| 12 December 2018 | Phil’s Stock World | ↻ Those designer babies everyone is freaking out about — it’s not likely to happen |
| 12 December 2018 | Business Daily | ↻ Those designer babies everyone is freaking out about — it’s not likely to happen |
| 14 December 2018 | Bizcommunity | ↻ Those designer babies everyone is freaking out about — it’s not likely to happen |
| 19 November 2018 | The New European | ↻ The truth behind the designer baby myth |
| 27 November 2018 | Pacific Standard | — The genetic case against designer babies |
| 3 January 2019 | Futurezone | Diese 7 krassen Dinge sind mit deinen DNA-Daten möglich |
| 3 January 2019 | Newsroom | CRISPR and the eternal sunshine debate |
| 4 January 2019 | NPO Radio 1 | Op zoek naar een beter embryo |
| 14 November 2018 | The Economist | Baby steps: A slippery slope towards designer babies? |
| 27 November 2018 | The Australian | ↻ Conceived with health in mind |
| 20 December 2018 | Trends/Tendances | ↻ Des séquences lourdes de conséquences |
| 10 January 2019 | Courrier International | ↻ Des séquences lourdes de conséquences |
| 17 January 2019 | The Courrier International | ↻ Bientôt, des bébés sélectionnés au gène près |
| 15 January 2019 | Christian Headlines | Babies made-to-order: America’s fast-food embryo industry |
| 20 January 2019 | The Christian Post | ↻ Babies made-to-order: America’s fast-food embryo industry |
| 21 January 2019 | Genetic Literacy Project | DNA as ‘fortune teller’? There are limits as to what genes can predict |
| 31 January 2019 | PR Newswire | Genomic Prediction Raises $4.5M in Sale of Preferred Stock to Support Innovation and Growth |
| 31 January 2019 | Contify Life Science News | ↻ Genomic Prediction Raises $4.5M in Sale of Preferred Stock to Support Innovation and Growth |
| 31 January 2019 | Middle East and North Africa Financial Network | ↻ Genomic Prediction Raises $4.5M in Sale of Preferred Stock to Support Innovation and Growth |
| 31 January 2019 | Business Wire | ↻ Genomic Prediction Raises $4.5M in Sale of Preferred Stock to Support Innovation and Growth |
| 1 February 2019 | Plus Company Updates | ↻ Genomic Prediction Raises $4.5M in Sale of Preferred Stock to Support Innovation and Growth |
| 12 February 2019 | STAT | Embryo editing for higher IQ is a fantasy. Embryo profiling for it is almost here |
| 16 February 2019 | BioEdge | Embryo profiling could be a short-cut to ‘designer babies’ |
| 22 February 2019 | La Tribune Hebdomadaire | La quête du surhomme à l'ère de CRISPR-Cas9 |
| 25 February 2019 | Genetic Literacy Project | ‘Genome profiling’—not gene editing—could offer easiest path to smarter babies |
| 16 March 2019 | The Sydney Morning Herald | Risk and reward: DNA tests alone shouldn’t make our biggest decision |
| 1 April 2019 | Genetic Engineering & Biotechnology News | Polygenic Risk Scores and Genomic Prediction: Q&A with Stephen Hsu |
| 1 April 2019 | Genetic Engineering & Biotechnology News | The Risky Business of Embryo Selection |
| 29 April 2019 | The Economist | The genomics revolution for good and ill |
| 8 May 2019 | Harvard Business Review | AI and the Genetic Revolution |
| 14 May 2019 | Next Big Future | Disease Risk Prediction from AI and Advanced Genomic Analysis |
| 23 May 2019 | Patheos | Bioethics Must Outpace Technology |
| 24 May 2019 | The Guardian | IVF couples could be able to choose the ‘smartest’ embryo |
| 25 May 2019 | Yerepouni Daily News | ↻ IVF couples could be able to choose the ‘smartest’ embryo |
| 24 May 2019 | Daily Mail | Couples undergoing IVF will be able to pick the ‘smartest’ embryo within 10 years, controversial US scientist says |
| 28 May 2019 | The Asian Age | Modification of the embryos’ IQ to soon be a reality |
| 28 May 2019 | Deccan Chronicle | ↻ Modification of the embryos’ IQ to soon be a reality |
| 28 May 2019 | America The Jesuit Review | Why don’t pro-life and disability rights groups work togeth­er more often? |

Supplementary Table S2. Quotes from Theme 1: A Slippery Slope Towards Designer Babies

| **Subtheme** | **Example quote** |
| --- | --- |
| Concerns about the slippery slope | Q1: “[T]here have been concerns that allowing parents to control some aspects of their child could be a slippery slope towards parents one day ruling out traits which don’t affect the child’s health, like hair or eye color.” (Daily Mail, 15 November 2018) |
| Concerns about eugenics | Q2: “Some of the listed conditions, even if [they are] diseases, are sliding into eugenics – not health” [Arthur L. Caplan, Professor of Bioethics, NYU School of Medicine] (Genetic Engineering & Biotechnology News, 1 April 2019).  Q3: “As geneticists further unravel the genome, the line between side-stepping disease and choosing genetic enhancement will thin out. We are sliding into Gattaca territory, in which successive generations are selected not only for health but also for beauty, intellect, stature and other aptitudes.” (Financial Times, 22 November 2018) |
| Reassurance about the misuse of PGT-P | Q4: “The company is only offering risk predictions involving disease and has no plans to predict an embryo’s eye color or level of educational attainment” [Nathan Treff, co-founder of Genomic Prediction] (The Wall Street Journal, 3 October 2018).  Q5: “<…> the company should not get too far out ahead of what society is comfortable with…we want there to be a broad discussion in society about what people think is appropriate” [Stephen Hsu, co-founder of Genomic Prediction] (Genetic Engineering & Biotechnology news, 1 April 2019) |
| Recurring argument | Q6: “For 50 years we have fretted about designer babies every time there is a new reproductive technology: mitochondrial donation and cloning were the most recent reason for dusting off the old canard.” (The Spectator, 16 December 2017)  Q7: “The designer babies have thus been called “future-we-should-not-want” for each new reproductive technology or intervention. But the babies never came and are nowhere close.” [Cecile Janssens, Professor of Epidemiology, Emory University] (The Conversation, 10 December 2018) |

Supplementary Table S3. Quotes from Theme 2: Well-being of the Child and Parents

| **Subtheme** | **Example quote** |
| --- | --- |
| Positive impact on well-being of the future child | Q1: “This screening reduces disease incidence, protecting health from the start: in-embryo.” (PR Newswire, 31 January 2019)  Q2: “Rather, it [the company] will read an embryo’s genes only to flag up potentially serious disorders, such as learning disabilities, dwarfism, diabetes and heart disease.” (Financial Time, 22 November 2018) |
| Positive impact on well-being of the parents | Q3: “[E]xperts said those tests can lead to a better chance of a successful pregnancy, attracting not only women over 35, but also men and women who are worried that their family history of diseases could be passed to their children.” (USA Today Network, 9 October 2018)  Q4: “With the rise of human genetic sequencing in 2003, scientists gained the ability to screen IVF embryos that carried a high risk for a debilitating disease, sparing parents from having to raise a child with, say, a heart defect or a mental illness.” (Outer Places, 3 December 2018) |
| Moral duty to improve quality of life of future child | Q5: “[I]f we accept that parents have an obligation to promote their children’s quality of life we should also allow them to use the existing technology of pre-implantation genetic profiling/diagnosis to select embryos for IVF implantation with genes associated with good physical and mental health” [Nicholas Agar, Professor in Philosophy, Victoria University of Wellington] (Newsroom, 3 January 2019) |
| Concern about well-being of the child | Q6: “Imagine, though, the misplaced burden of expectation on a child “selected” to be bright who doesn’t live up to it. If embryo selection for high IQ goes ahead, this will happen.” (The Guardian, 19 November 2018) |
| Pressure to use artificial reproduction | Q7: “When the technique becomes widely available, as it no doubt will, those wealthy enough to do so may opt to undergo IVF even if they are able to conceive naturally.” (The Economist, 14 November 2018)  Q8: “Hsu’s prediction is that “billionaires and Silicon Valley types” will be the early adopters of embryo selection technology, becoming among the first “to do IVF even though they don’t need IVF.” (MIT Technology Review, 1 November, 2017) |

Supplementary Table S4. Quotes from Theme 3: Impact on Society

| **Subtheme** | **Example quote** |
| --- | --- |
| Socio-economic impact | Q1: “In the future, we may have huge customers from another country, and they are really demanding that they want to be able to do cosmetics. They really want to know who has lighter colored skin and darker skin. It will be a tough decision for us. If they are ordering 100,000 tests from us, and they really want this feature—which we can do, and which is 100% legal in South Korea—what are we going to do? So, I’m not going to prejudge what we are going to do” [Stephen Hsu, co-founder of Genomic Prediction] (Genetic Engineering & Biotechnology news, 1 April 2019) |
| Issues of discrimination and equality | Q2: “One or two babies in every hundred are born with genetic disease; millions inherit higher risks of cancer and dementia. Set against that is the grim specter of a world in which part of the race is biologically self-improving, while leaving behind the genetic have-nots.” (The Times, 17 November 2018) |
| Attitude of society towards disabled people | Q3: “Under the context of giving your child “the best possible start”,  wouldn't we risk removing the difference? <…> Einstein, Newton, and  all these geniuses who suffered from Asperger syndrome - would they  be the same, without that?” (C Net France, 14 December 2017) |
| Individual rights vs needs of society | Q4: “Most parents would feel that choosing an embryo likely to produce a more intellectually able child was securing the welfare of that child, despite creating a societal division with another child for whom that choice was unavailable.” (The Times, 17 November 2018) |

Supplementary Table S5. Quotes from Theme 4: Deliberate Choice

| **Subtheme** | **Example quote** |
| --- | --- |
| Positive impact on deliberate choice | Q1: “Many [...] parents using IVF are confronted by an “embryo choice”  problem: They have more viable embryos than they intend to use. For  these parents, it is useful to have additional information about each  embryo, such as whether it is at high risk for certain health conditions.” (Psychology Today, 2 December 2018)  Q2: “Since fertility treatment often produces multiple viable embryos,  only one or two of which can be implanted, prospective parents could  pick those with the “best” genes.” (Financial Times, 22 November 2018) |
| What lives are worth living? | Q3: “Once you do these kinds of things, you literally are having impact on what kind of people get created and what kind of people exist in the world.” [Kimberly Mutcherson, Professor of Law, Rutgers Law School] (USA Today Network, 9 October 2018) |
| Concern about deliberate choice | Q4: “Parents may even think it’s their moral duty to choose the “best” possible baby, not just for themselves but to serve the national interest.” (Financial Times, 22 November 2018)  Q5: “An IVF doctor has two healthy, viable embryos and must choose,  which to implant. One has a hypothetical risk score that indicates the embryo is at high risk for struggling academically in school. The second embryo has a score indicating the future child likely won’t struggle. Do you tell the parents?” (The Wall Street Journal, 3 October 2018) |

Supplementary Table S6. Quotes from Theme 5: Societal Readiness

| **Subtheme** | **Example quote** |
| --- | --- |
| Concerns about unregulated commercialization | Q1: “[E]ven though complex traits such as intelligence, athletics and musicality cannot be selected or designed, there will be opportunists who will try to offer these traits, even if totally premature and unsupported by science. <…> People need to be protected against this irresponsible and unethical use of DNA testing and editing [Cecile Janssens, Professor of Epidemiology, Emory University] (The Conversation, 10 December 2018)  Q2: “Geneticist Peter Visscher likened the test to “cloning a dead pet,” in that people would “pay hundreds of thousands” without actually understanding what the technology does”. (Forbes, 1 December 2018)  Q3: “Embryo selection needs robust regulation that society can be confident in. <…> Leaving a matter such as this to unregulated market forces is dangerous.” [Ewan Birney, Director of European Bioinformatics Institute - EMBL-EBI] (The Guardian, 19 November 2018) |
| Lack of scientific validity | Q4: “The obstacle to polygenic tests has been that with so few cells, it’s been difficult to get the broad, accurate view of an embryo’s genome necessary to perform the needed calculations.” (MIT Technology Review, 1 November 2017)  Q5: “Polygenic traits are complicated: if you have 100 different genes in a trait, keeping slightly better versions of 10 of them isn’t going to get you very much.” [Henry T. Greely, Professor of Law, Stanford Law School] (Genetic Engineering & Biotechnology news, 1 April 2019).  Q6: “Hundreds or thousands of particular alleles influence the ultimate cognitive capacity of a human, along with the facts of his uterine development and early life (National Post, 16 November 2018) |
| Drawback of polygenic risk scoring | Q7: “PRS are typically used to stratify a population into segments, distinguishing the clinically meaningful changes that occur at the two tail ends. The test is meant to “drop people into broad buckets,” not to make an accurate individual prediction.” [Laura Hercher, Director of Research, Human Genetics, Sarah Lawrence College] (Genetic Engineering & Biotechnology News, 1 April 2019)  Q8: “Most of the genomes that have been scanned are of people of European descent. This means that results may be less reliable for Africans, Chinese and Indians.” (NPO Radio 1, 4 January 2019) |
| Unwanted consequences | Q9: “[T]here are concerns the genes for desirable traits—like high intelligence—may also cause unwanted effects which scientists don’t know about. For example, past research has linked those genes to a higher risk of having autism.” (Daily Mail, 15 November 2018) |
